# A mixed-methods study on the implementation of a mobile health application (mHealth app) for stroke caregivers in Malaysia: healthcare providers’ perspective

**DOI:** 10.3389/fneur.2023.1222260

**Published:** 2023-10-12

**Authors:** Norsima Nazifah Sidek, Tengku Alina Tengku Ismail, Sureshkumar Kamalakannan, Xin Wee Chen, Muhammad Hibatullah Romli, Mohamad Zarudin Mat Said, Iliatha Papachristou Nadal, Khairul Azmi Ibrahim, Kamarul Imran Musa

**Affiliations:** ^1^Department of Community Medicine, School of Medical Sciences, Universiti Sains Malaysia, Kota Bharu, Kelantan, Malaysia; ^2^Clinical Research Centre, Hospital Sultanah Nur Zahirah, Ministry of Health Malaysia, Kuala Terengganu, Terengganu, Malaysia; ^3^Department of Non-Communicable Disease Epidemiology, London School of Hygiene and Tropical Medicine, London, United Kingdom; ^4^Department of Social Work, Education, and Community Well-being, Northumbria University, Coach Lane Campus, Newcastle upon Tyne, United Kingdom; ^5^Department of Public Health Medicine, Faculty of Medicine, Universiti Teknologi MARA, Sungai Buloh, Selangor, Malaysia; ^6^Department of Rehabilitation Medicine, UPM Teaching Hospital, Faculty of Medicine and Health Sciences, Malaysian Research Institute on Ageing (MyAgeing™), Universiti Putra Malaysia, Serdang, Selangor, Malaysia; ^7^Disease Control Unit, Hilir Perak District Health Office, Teluk Intan, Perak, Malaysia; ^8^Division of Care in Long Term Conditions, King’s College London, London, United Kingdom; ^9^Department of Medicine, Hospital Sultanah Nur Zahirah, Ministry of Health Malaysia, Kuala Terengganu, Terengganu, Malaysia

**Keywords:** mHealth app, stroke caregivers, mixed method, stroke healthcare provider, acceptability

## Abstract

**Introduction:**

Recognizing the burden experienced by caregivers of stroke survivors, an intervention using mobile health applications (mHealth apps) has been proposed to support and empower stroke caregivers. This study aimed to assess the acceptability and expectations of healthcare providers, who play a vital role as gatekeepers in the healthcare system, to ensure the effectiveness and sustainability of the intervention.

**Methods:**

This was a concurrent mixed-method study design, with healthcare providers involved in stroke care management in the northeast regions of Malaysia as study participants. The qualitative component of the study was conducted using a phenomenological approach that involved in-depth interviews to explore the acceptability and expectations of healthcare providers regarding the adoption of mHealth apps in the context of stroke caregiving. The study was complemented by quantitative data collected through an online survey using an adjusted version of the technology acceptance model tool.

**Results:**

In total, 239 participants from diverse backgrounds and professions were enrolled in the study, with 12 in the qualitative component and 227 in the quantitative component. The findings from the quantitative survey showed that over 80% of the participants expressed their intention to use mHealth apps. The qualitative component generated two themes related to the acceptability and expectations of mHealth apps, which were integrated with the quantitative findings. Additionally, in-depth interviews revealed a new theme, namely the key features of mHealth, with three sub-themes: availability of services for caregivers, provision of knowledge skills, and supporting caregivers in managing stroke patients.

**Conclusion:**

Healthcare providers demonstrated excellent acceptability of this mHealth intervention as part of caregiving assistance, particularly with the inclusion of essential key features. However, future investigations are necessary to establish the feasibility of integrating the mHealth app into the healthcare system and to ensure its long-term sustainability.

## 1. Introduction

Stroke has become a leading cause of preventable disability, and the burden has increased worldwide over the past two decades (1). In 2017, about one in every two Malaysian stroke survivors was discharged with physical disabilities (2) and required aid and extensive care to adjust to their new life. These include performing activities of daily living (ADLs), rehabilitation support, medication care, companionship, and emotional support (3, 4).

The World Health Organization (WHO) Global Observatory for eHealth defined mHealth apps as mobile applications and other wearable devices that collect and monitor users’ medical information (5). Studies have indicated that it enhances the excellence and inclusivity of healthcare; expands access to health-related information, services, and expertise; and fosters improved health-related behaviors (6–8). With limitations in the health system, such as human resources, infrastructure, financial support, and the caregivers themselves (including a lack of social support, logistical issues, and geographic factors), this mHealth intervention seems to bring forth a promising solution in stroke caregiving (9–13).

Numerous non-pharmacological treatment modalities, such as psychoeducational and psychosocial information, and skill-building interventions have been adopted and delivered on various digital platforms (including mobile health applications) to assist and empower stroke caregivers (14–17). A scoping review of seven articles regarding mHealth apps for family caregivers in preventing recurrent stroke revealed that many apps used video education, exercise reminders, access to stroke-related information, and feedback mechanisms (17). The studies were conducted in seven countries, namely the United States, Pakistan, the Netherlands, Korea, India, China, and Sweden, and all studies were conducted to test the feasibility of the apps in question (17). Another review of 47 apps that were available on the Android and/or iOS platforms and tailored to caregivers during stroke patient recovery revealed that such apps contained features that support caregivers’ wellbeing, role adaptation, and engagement in patient management (18). The sources or contents of the apps were derived from Australia, Canada, India, Singapore, Spain, and the United States. However, some of them could not be identified.

Due to the unavailability of an app that is culturally tailored to Malaysia and specific for stroke caregivers, a megaproject was launched in 2019 through a collaboration between Universiti Sains Malaysia and the London School of Hygiene and Tropical Medicine (LSHTM) (19), with the aim of developing an app that can help enhance the caregivers’ ability to handle the physical and psychological requirements of stroke survivors, as well as their own personal needs (19). This project was based on the success of an application known as “Care for Stroke,” a smartphone-enabled intervention for stroke patients that was tested in India and was regarded as acceptable by more than half of stroke survivors and 90% of caregivers (20).

Nevertheless, prior to developing an mHealth app, it is crucial to explore the acceptability of healthcare providers and their expectations of this intervention. The concept of “acceptability” was employed to refer to how users perceive a system prior to its use (21, 22). Healthcare providers who are responsible for providing stroke care and engaging with both patients and caregivers are in a prime position to introduce this mHealth app during their regular duties. The perspective that healthcare providers hold toward this technology could significantly impact the willingness of patients and caregivers to adopt it (23–25). Furthermore, the reasons for low mHealth app usage rates include healthcare providers’ perspectives, acceptability, and over-reliance on traditional face-to-face interventions (25, 26). Overall, this created a need to explore the acceptability of healthcare providers toward adopting digital health interventions prior to app development.

Apart from concerns regarding the suitability and adaptability of the app’s contents to the local context, most studies have applied quantitative methods to assess the acceptability aspect (21, 22, 27, 28). Acceptability should be researched using a variety of methods, combining specific, detailed insights from qualitative research, which is inclined to be based on interpretivism, with generalizable, easily replicable data from quantitative research supported by positivism (27). To bridge these gaps and yield more credible results, this study aimed to present mixed-method research to explore the acceptability of healthcare providers for the implementation of an mHealth app in stroke care. The quantitative component measured mHealth acceptability, while the qualitative component deeply explored the perception and expectations of stroke healthcare providers toward mHealth. The extent to which stroke healthcare providers’ acceptability toward the mHealth app supported or contradicted their perceptions and expectations was observed. It is hoped that the results could provide a piece of holistic evidence by combining inductive and deductive thinking.

## 2. Methodology

### 2.1. Study design

The study was conducted based on a pragmatic paradigm, which aims to comprehensively answer the research questions by applying the most effective methods to solve practical issues in the real world, allowing the use of various data sources and knowledge (28). A concurrent mixed-methods design was applied to align with this approach, with quantitative and qualitative data collected simultaneously within the same timeframe (29).

### 2.2. Study setting and study population

This study was conducted in Malaysia’s northeast coastal regions between 1 June 2021 and 12 November 2021. Our study sample consisted of healthcare providers, such as neurologists, rehabilitation physicians, nurses, occupational therapists (OT), and physiotherapists (PT), from various government health sector facilities, including tertiary hospitals, district hospitals, and primary care facilities. Only the healthcare providers with at least one year of experience managing stroke survivors were included in this study.

To obtain an appropriate sample size for the qualitative component, the principle of saturation was implemented, which meant conducting interviews until no new information or perspective could be obtained (29, 30). Based on a previous study, the required number of participants ranged from 10 to 24 (31–33). To ensure a range of perspectives, experiences, and viewpoints, participants in the interviews were enrolled purposefully using the maximum variation sampling (different professions, ages, sexes, and facility settings) technique (34).

For the quantitative component, sample size estimation was performed using the Krejcie and Morgan formula (35), with the total number of occupational therapists and physiotherapists reported as 279 (36). The total number of physicians, including neurologists, rehabilitation physicians, medical officers, and rehabilitation nurses, based on the staff registry in the respective facilities, was approximately 80. Hence, the minimum sample size required was 186 participants, involving 156 therapists and 30 other stroke healthcare providers.

### 2.3. Study procedures

#### 2.3.1. Qualitative data collection and sampling

This component employed semi-structured individual interviews with an interview guide as the research instrument (Table 1) (37). Two pilot interviews, which were not included in the sample, were conducted to refine the interview guide and gain deeper insight into the procedure.

**Table 1:** Topics for the in-depth interviews.

**Mobile apps development for stroke caregiving** We are planning to develop a mobile app specifically to assist stroke caregivers. What do you think about this idea? What do you expect from the apps? What, in your opinion, is the most crucial aspect of the app’s functionality? How would you describe your intention to support the implementation of this app? How about the caregiver? How do you think their acceptance toward the usage of this app?

For participant recruitment, the snowball sampling method was used (38), whereby the names of those eligible and interested in participating in the interview were obtained from the head of the respective profession and contacted by phone. The interviews were planned to be conducted in person, but due to the unforeseeable outbreak of the COVID-19 pandemic and the implementation of associated mitigation measures as well as logistical difficulties related to conducting face-to-face interviews, the interviews were performed using telephone or video conferencing via Webex at the participants’ convenience. Interviews were conducted either in Malay or English language, depending on the participants’ preferences. Digitally recorded interviews were transcribed verbatim in their original languages. Participants were assigned identifying codes to ensure privacy and data management. The audio-recorded interviews and transcribed data were encrypted and stored securely with only the first author accessing them.

To establish trustworthiness, the following four criteria were proposed by Lincoln and Guba: credibility, confirmability, dependability, and transferability (39). The research team ensured credibility by constantly reviewing the results, ensuring sufficient time was allocated for data acquisition, and engaging in research for even longer periods. Confirmability was obtained by recruiting experienced stroke treatment healthcare professionals, and all research features were thoroughly reviewed to meet transferability requirements (34). Member checking was employed to validate the results by discussing the findings with selected participants to guarantee their correctness in reflecting their perspectives and experiences (34). Furthermore, the investigator triangulation method was used to remove researcher bias by comparing preliminary interpretations and findings with the original data (34).

#### 2.3.2. Quantitative data collection and sampling

For this component, to ensure the clarity and comprehension of questionnaires, face validity was conducted before the data collection process, involving 10 healthcare providers, namely 3 physicians, 5 therapists, and 2 nurses. These individuals were excluded from this study. Based on the ratings, the questionnaire had good overall face validity (S-FVI/UA = 0.88; S-FVI/Ave = 0.99). Some questions were rephrased based on expert opinions and their applicability to stroke healthcare providers.

Subsequently, a cross-sectional study was conducted using a structured questionnaire. The questionnaire consisted of 2 sections and 23 questions. The first section contained nine items, organized as follows: (i) sociodemographic profile, including the aspects of sex, age, profession, highest education level, place of work, state, and work experience in stroke management; (ii) self-reported internet skill, in which participants were asked if they could use the Internet for a daily purpose, such as checking and replying to an email, browsing the Internet, and searching for information, which were coded as “very good,” “good,” “average,” “fair,” and “poor”; and (iii) availability and usage of technology at their workplace. The selections for technology usage items were designed based on the technology available in Malaysia: electronic patient records, online self-management or treatment modules, online patient files (via a secure portal), making online appointments, a website for client information (e.g., downloading resources from the Ministry of Health portal), online training, electronic/online screening, eConsult (secure email contact with the client), telemedicine (video calling and remote care), Hospital Information System (HIS), and others. The research team developed sociodemographic and job-related factors based on relevant factors that may impact the acceptability of mHealth as well as information from previous studies (40, 41).

The second section contained the adjusted version of the technology acceptance model (TAM) questionnaire, which measures the intention of healthcare providers to adopt and support mHealth app usage, adapted with the author’s permission (40). The questionnaire was designed based on the core constructs of TAM (42). This 14-item questionnaire assessed perceived usefulness (6 items), perceived ease of use (6 items), and intention to use (2 items). Each item was scored on a 7-point Likert scale, ranging from 1 (strongly disagree) to 7 (strongly agree), with 4 as the neutral center of the range.

The convenience sample and snowballing strategy were implemented with the help of each profession’s head and liaison officer to identify eligible participants (38). This cross-sectional, anonymous survey was conducted online using Google Form^®^, a cloud-based survey tool, and shared through email and WhatsApp^®^ to overcome geographical limitations and restrict physical contact considering the COVID-19 outbreak.

Once all responses were collected, a psychometric evaluation of the questionnaire’s internal structure was performed to ensure its validity and reliability. Internal consistency and factorial structure were examined using exploratory factor analysis (EFA), which showed that the data were normally distributed. The sampling adequacy was excellent, as evidenced by the statistically significant Bartlett’s test and KMO value of 0.953. The 14 items were subjected to a principal component analysis, which revealed an apparent factor structure (Supplementary Table S1). The varimax schedule-rotated component matrix indicated satisfactory loading of all items on their corresponding factors, accounting for 88.88% of the variation in the dependent variable (35.79, 35.02, and 18.07% for factors 1, 2, and 3, respectively). None of the items required deletions in the EFA model.

As for the internal consistency and reliability, Cronbach’s alpha ranged between 0.965 and 0.975. These results indicated that the questionnaire used in this study was valid and reliable for measuring the acceptability of mHealth among healthcare providers.

### 2.4. Data analyses and integration technique

For the qualitative component, the qualitative data analysis software NVivo^®^ for Windows (QSR International, release 1.5.2, 2021) was used for qualitative data management. All transcribed audio recordings were exported to software and processed using inductive theme analysis (43). The process of data analysis entailed becoming acquainted with the data, creating initial codes, consolidating codes into themes, and assessing, defining, naming, and reporting themes (43). The first, second, and third authors were responsible for the data coding and qualitative data analysis. Any coding disagreements were discussed and settled by consensus within the study team.

Regarding the quantitative component, questionnaires were retrieved from Google Form^®^ and inserted into the IBM SPSS^®^ version 26.0 software for Windows (IBM Corp., Version 26.0, Armonk, NY) for analysis. Descriptive data were reported as frequency (proportion) for categorical data, and mean (standard deviation, SD) was used for numerical data. The Student *t*-test and Fisher-exact test were used to compare the participants’ characteristics between the qualitative and quantitative components. Meanwhile, to look for possible individual variations in the quantitative component, the Student *t*-test or one-way ANOVA was applied, depending on the variables. A *p*-value of less than 0.05 was considered statistically significant.

After conducting the primary data analysis, the research team discussed the possibility of combining the two datasets. A convergent design was employed to explore healthcare providers’ acceptability and expectations concerning mHealth more thoroughly. Given that the quantitative scales and initial qualitative interview questions about acceptability were administered concurrently, this approach allowed for more comprehensive data analysis. A narrative weaving approach was applied to describe and merge the outcomes of the first part by reporting the findings of both components together on a theme-based basis (44). The joint display technique was then utilized to present quantitative and qualitative data together for easy comparison and interpretation (29).

### 2.5. Ethical considerations

The study was registered and approved by all responsible ethics committees (Malaysia Research Ethics Committee, MREC Ref: KKM/NIHSEC/P20-922), the Human Research Ethics Committee of USM (JEPeM: USM/JEPeM/20010031), and the London School of Hygiene & Tropical Medicine (LSHTM) Research Ethics Committee (LSHTM Ethics Ref: 19079). Regarding the qualitative aspect of the study, participants were given a detailed explanation, both orally and in writing, about the study’s objectives, their voluntary participation, and the secure management of their audio recordings. Following the data collection, all interview transcripts were promptly anonymized. Every participant provided consent to participate in the study and to record their interviews, both verbally and in writing. Compensation of RM 50 was offered to the participants for their time. For the quantitative component, the online survey incorporated the consent form, and individuals who did not provide their consent were unable to proceed to the survey section. Compensation of RM 20 was provided to the participants.

## 3. Results

### 3.1. Descriptive summary of respondents’ background information

Table 2 presents the characteristics of participants in both components, whereby both groups were comparable with no significant differences in age, sex, or working experience. For the qualitative component, 12 healthcare providers were interviewed for an average of 30 min. Meanwhile, for the quantitative component, there were a total of 265 healthcare providers who participated in the survey, but 38 responses had to be discarded due to insufficient information. Thus, a total of 227 participants with a mean (SD) age of 34.9 (6.36) years were included in the quantitative analysis. Most participants (*n* = 173, 76.2%) were women, with half of them working as physiotherapists at hospitals (either tertiary or district), with a mean (SD) working experience in stroke care of 7.0 (4.95) years.

**Table 2:** Participant characteristics.

Variable	Qualitative component (*n* = 12)	Quantitative component (*n* = 227)	*p*-value
Sex			
Female	8 (66.7%)	173(76.2%)	0.492*
Male	4 (33.3%)	54(23.8%)
Age			
Mean (SD) year old	38.1 (8.8)	34.9	0.232**
≤30	4 (33.3%)	65 (28.6%)
31–40	4 (33.3%)	118 (52.0%)
≥41	4 (33.3 %)	44 (19.4%)
Working experience in stroke care			
Mean (SD) years	5.7 (3.7)	7.0 (4.95)	0.397**
1–5	6 (50.0%)	97 (42.7%)	
>5	6 (50.0%)	130 (57.3%)	
Profession			
Physiotherapist	3 (25.0%)	119 (52.4%)	–
Occupational therapist	3 (25.0%)	76 (33.5%)	
Nurse and medical assistant	3 (25.0%)	18 (7.9%)	
Physician	2 (16.7%)	10 (4.4%)	
Pharmacist	0	4 (1.8%)	
Home manager	1 (8.3%)	0	
Working place			
State hospital	5 (41.7%)	88 (38.8%)	–
District hospital	4 (33.3%)	36 (15.9%)	
Primary care center	2 (16.7%)	99 (43.6%)	
Home care and rehabilitation center	1 (8.3%)	4 (1.8%)	

*Fisher exact test was applied. **Student’s *t*-test was applied.

Assessment of any potential individual variance in the quantitative component was performed, and Table 3 represents the mean scores for each TAM domain for subgroups of the sample, based on sex, age group, profession, working experience group, working place, and internet skills. The analysis showed no significant differences between all respondent characteristics for domain perceived ease of use. A similar outcome was observed for perceived usefulness except for profession, but further analysis using the Bonferroni-corrected post hoc revealed no significant difference between any groups. With regards to intention to use; profession, working experience, and internet skills were found to influence the intention to use the mhealth app significantly.

**Table 3:** Mean score analysis for each TAM domain by the subgroups of the study participants, based on sex, age group, profession, working experience group, working place, and internet skill (*n*=227).

**Characteristic**	***n***	**Perceived usefulness**	***p*-value**	**Perceived ease of use**	***p*-value**	**Intention to use**	***p*-value**
**Sex** Male Female	54 173	5.3(1.52) 5.5(1.34)	0.560*	5.0(1.20) 5.1(1.26)	0.591*	5.7(1.46) 5.6(1.35)	0.643*
**Age group** ≤30 31-40 ≥41	65 118 44	5.4(1.31) 5.5(1.43) 5.3(1.39)	0.867**	5.2(1.18) 5.1(1.26) 5.0(1.29)	0.600**	5.7(1.33) 5.7(1.39) 5.4(1.41)	0.538**
**Profession** Physiotherapist Occupational therapist Nurse and Medical Assistant Physician Pharmacist	119 76 18 10 4	5.3(1.40) 5.9(1.32) 5.7(1.33) 6.2(1.03) 7.0(0.00)	0.039**	5.0(1.24) 5.2(1.23) 5.3(1.30) 5.5(1.12) 6.4(0.59)	0.076**	5.3(1.40) 5.9(1.32) 5.7(1.33) 6.2(1.03) 7.0(0.00)	0.008**
**Working experience** 1-5 years >5 years	97 130	5.5(1.37) 5.3(1.39)	0.273*	5.2(1.13) 5.0(1.31)	0.175*	5.8(1.30) 5.5(1.41)	0.044*
**Working place** State Hospital District Hospital Primary care centre Home care and Rehab Centre	88 36 99 4	5.4(1.38) 5.5(1.51) 5.4(1.22) 5.9(1.07)	0.827**	5.0(1.31) 5.2(1.31) 5.1(1.17) 5.8(0.88)	0.575**	5.7(1.41) 5.6(1.62) 5.5(1.25) 6.5(1.00)	0.444**
**Internet skill** Poor and Fair Average Good Very good	3 52 102 70	4.7(2.75) 5.1(1.35) 5.5(1.12) 5.6(1.65)	0.112**	4.4(2.58) 4.8(1.26) 5.1(1.04) 5.3(1.40)	0.153**	5.2(1.50) 4.2(2.75) 5.7(1.12) 6.0(1.46)	0.004**

*Student *t*-test was applied, **One way Anova test was applied.

### 3.2. mHealth apps acceptability and expectation

Regarding the “Stroke” mHealth app acceptability, Table 4 illustrates the joint display of both components with four key findings: (i) QUAL analysis revealed three subthemes related to the TAM model (perceived usefulness, perceived ease of use, and intention to use); (ii) three-quarters of respondents agreed that the “Stroke” mHealth app is perceived to be useful; (iii) two-quarters of respondents agreed that the “Stroke” mHealth app is perceived as easy to use and welcome using it; and (iv) more than four-fifths have the intention to use the “Stroke” mHealth app.

**Table 4:** Joint display of healthcare provider acceptability toward “Stroke” mHealth app.

Overarching theme	Categories	Qualitative findings		Quantitative findings						
			Item	Number of answer, n (%)						
				Strongly disagree	Moderately disagree	Somewhat disagree	Neutral	Somewhat agree	Moderately agree	Strongly agree
MHealth app acceptability and expectation	Perceived usefulness	*Yeah, I think it will be very helpful. Both to clinicians, to caregivers, and also to the care, the health providers. (Neurologist)* *Aaah, it saves time and in terms of explanation, it will be more uniform (OT 1)* *When there is a video like this, we can just show it, it means that the interview session will be shorter, and we have more time to cater to other patients. (OT 1)* *If this mobile app is available, I think it will be very helpful in terms of the treatment of stroke patients. For the caregivers and the stroke patients themselves. (OT 2)*	1. Improves the care	8 (3.5)	9 (3.9)	4 (1.7)	40 (17.3)	35 (15.2)	72 (31.2)	63 (27.3)
			2. More productive	10 (4.3)	7 (3.0)	4 (1.7)	37 (16.0)	53 (22.9)	65 (28.1)	55 (23.8)
			3. More effective	8 (3.5)	8 (3.5)	10 (4.3)	33 (14.3)	45 (19.5)	69 (29.9)	58 (25.1)
			4. Beneficial to job	7 (3.0)	8 (3.5)	1 (0.4)	31 (13.4)	49 (21.2)	67 (29.0)	68 (29.5)
			5. Provide care to patients more quickly	7 (3.0)	10 (4.3)	2 (0.9)	29 (12.6)	51 (22.1)	72 (31.2)	60 (26.0)
			6. Easier to provide care to patients	5 (2.2)	10 (4.3)	2 (0.9)	30 (13.0)	43 (18.6)	85 (36.8)	56 (24.2)
	Perceived ease of use	*I think very important if it is available in multiple languages because you know, in Malaysia we have different ethnics. (Neurologist)* *It must be easy to use. User friendly. (Neurologist)* *When you are access the apps, it’s so easy. It supposed to be easily accessible (PT 1) [SIC]* *Yes, the keyword is easy to understand (PT 3)* *I think that if the app is user-friendly and is, rich in content and content and information. I think it should be an eye-opener (PT 2)*	7. Clear and understandable	2 (0.9)	11 (4.8)	9 (3.9)	51 (22.1)	48 (20.8)	72 (31.2)	38 (16.5)
			8. Requires little effort for me	3 (1.3)	11 (4.8)	9 (3.9)	66 (28.6)	44 (19.0)	67 (29.0)	31 (13.4)
			9. Easy to use	1 (0.4)	12 (5.2)	4 (1.7)	72 (31.2)	43 (18.6)	65 (28.1)	34 (14.7)
			10. Easily use for what users want it to do	2 (0.9)	10 (4.3)	3 (1.3)	59 (25.5)	45 (19.5)	75 (32.5)	37 (16.0)
			11. Learning is easy	2 (0.9)	12 (5.2)	2 (0.9)	62 (26.8)	46 (19.9)	67 (29.0)	40 (17.3)
			12. Easy to become proficient	1 (0.4)	11 (4.8)	5 (2.2)	58 (25.1)	45 (19.5)	76 (32.9)	35 (15.2)
	Intention to use	*I’m happy if there are apps like that because right now, it can be said that almost everyone has a smartphone. (OT 2)* *Happy to uselah. Because, sometimes it’s easier, like you do not have to search on Google for everything (Nurse 3)*	13. Intend to use them	6 (2.6)	7 (3.0)	2 (0.9)	28 (12.1)	43 (18.6)	73 (31.6)	72 (31.2)
			14. Predict would use it	4 (1.7)	8 (3.5)	4 (1.7)	29 (12.6)	42 (18.2)	72 (31.2)	72 (31.2)

### 3.3. Key Features of mHealth App

This section explains the expectations of healthcare providers that emerged from the qualitative component. The following three subthemes were developed: (i) availability of services for caregivers, (ii) provision of knowledge skills, and (iii) supporting caregivers in managing stroke patients. The details of the subthemes, codes, and quote examples are presented in Table 5.

**Table 5:** Subtheme and codes for key features of the mHealth for caregiving theme

Subtheme	Code	Quotes
Availability of services for caregivers	Support for emotional wellbeing Careline Directory of healthcare service and financial support	*For the caregivers to take care of themselves. Uhm, I think, uh. Most important is their, uh, their mental health (Nurse 2)* Because if we are looking into the care, into the caretaker burden, I believe that, uh, I believe that the questionnaire of DASS 21* can be included there, even as an outcome measure for us to monitor the patient. (PT 3) *It should provide the places closest to the patient that can provide service related to the stroke (OT 2)*
Provision of knowledge skill	Exercise for stroke patient Important of rehabilitation Role of caregiver Stroke disease and awareness Post-stroke management Stroke prevention strategy	*When we understand about the illness, its effects, the recovery process, it would be easier to take care of that person (Homecare manager)* *What to do to improve his condition, any symptoms, like hemiparetic, face paretic, dysarthria. For example, he has difficulty to talk… The specific therapy is already in the apps (Rehab physician)* *Ha, from what I see is that if you want to take care of stroke patients, you must know the techniques. For example, from an emotional point of view, how to persuade them, how to spend time with them (Homecare manager)*
Managing stroke patient assistance	Appointment scheduling Progress monitoring To-do list	*Most important from what I see is appointment scheduling for physio. So, we can use the apps not only to provide the details of the appointment, but for us to set link to the patient or caregiver phone as the reminder (PT 2)* *To monitor patient progress. No need to search for anywhere else, It means for Example bed mobility, we can monitor his current progress, then maybe In the form of a template, In the form of a video or perhaps a checklist for step-by-step instruction. (PT 3)*

*Depression Anxiety and Stress Scale 21.

#### 3.3.1. Availability of services for caregivers

The most common caregiver service addressed by participants was support for emotional wellbeing. A few methods suggested by the participants were linked to counseling therapy, peer support groups, respite care availability, and a spiritual and holistic approach.

The care line or helpdesk service was also felt to be necessary for caregivers who required urgent clarification or assistance in managing the patient. Finally, they requested the directory of healthcare-related services, such as emergency and ambulance directories, home care support, and physiotherapy services, to assist them should they require such support. To ease the burden on caregivers, a few participants believed that this app could be a decent platform for financial assistance by providing an external link to respective agencies.

#### 3.3.2. Provision of knowledge skill

Most respondents stressed the need to provide caregivers with information from a reliable and reputable source to help them adjust to their new responsibilities. The information suggested was about exercises for stroke patients, the importance of rehabilitation, the caregiver’s role, and education regarding stroke disease and awareness. A few contributors also highlighted post-stroke management, such as stroke complications, danger signs to monitor for stroke prevention strategies, and managing patient comorbidities. In addition, some believed that the approach or strategy for comprehending the patient’s emotions and how to manage them should be included in the app.

#### 3.3.3. Supporting caregivers in managing stroke patients

Appointment scheduling integration was one of the aspects highlighted by the participants. One participant expressed an issue that is commonly faced by caregivers, which is encountering difficulties in contacting the right department to reschedule an appointment, resulting in caregivers having to travel a long distance to schedule another appointment. Since the percentage of patients who defaulted on rehabilitation clinic follow-up was significant and there was insufficient staff to follow up with defaulters, appointment reminders were also frequently mentioned by participants.

Specifically, for the recovery process, some participants anticipated that the app would include progress monitoring. This functionality is valuable to healthcare providers for assessing and monitoring patients’ progress and is beneficial to patients with multiple caregivers. In addition, they proposed a reminder or to-do list to ensure that they do not forget the daily tasks that a stroke survivor must complete, such as rehabilitation exercises for stroke recovery.

## 4. Discussion

To the best of our knowledge, this is the first study to employ a mixed-methods research design to explore the acceptability and expectations of healthcare providers regarding mHealth app implementation aimed at supporting caregivers. In contrast to previous studies (21, 22, 40, 41), a comprehensive approach was adopted to establish a framework that was both generalizable and insightful (29).

Regarding individual variation in the quantitative component, in line with previous studies (25, 27), perceived usefulness, perceived ease of use, and intention to use among healthcare providers towards the mHealth app were not influenced by sex and age. However, professionals, particularly physicians and pharmacists, were more prone to use the app than their counterparts. This trend might be attributed to the increasing utilisation of mHealth apps in their daily routines (7, 25, 27). In addition, participants with more years of experience in stroke care were also shown to have greater intention towards implementing the app. With longer working experience, and being a firsthand witness, healthcare providers become more familiar with the challenges and complexities faced both by the stroke patient and their caregivers, as well as the unmet needs of caregivers over time (10, 13). These experiences can drive the intention to adopt innovative solutions like mHealth apps that have potential benefits to fill the gaps in support.

The present study revealed two important findings: (i) most healthcare providers accepted and supported the introduction of the mHealth app in assisting stroke caregivers, proven by the fact that more than 80% had the intention to use it, more than 70% perceived mHealth to be useful, and more than 60% perceived mHealth to be easy to use and (ii) the two themes derived from the qualitative method were mHealth app acceptability and expectation and the key features of mHealth for caregiving. Our findings will contribute to the existing body of knowledge on the gap between healthcare providers’ acceptability and expectations toward the implementation of mHealth apps in assisting stroke caregivers.

The high acceptability among Malaysian healthcare providers of mHealth applications in assisting stroke caregivers may stem from their potential to streamline healthcare delivery, alleviate the burden on healthcare providers, and improve public health outcomes through more direct and efficient intervention. This, in turn, can enhance the quality of care and align it with the expectations of the target community (24, 40, 45, 46). Several barriers to healthcare access during the recent COVID-19 pandemic, such as public anxiety that accessing a hospital might be unsafe, movement restrictions, and financial constraints, probably boosted healthcare providers’ acceptability of digital care interventions (13, 47). Acceptability is further reinforced by the adoption of a diverse range of digital technology and mobile health (mHealth) apps in their daily practices (25, 48). This is supported by the findings of the present study, which showed that two-thirds of the participants possessed proficient internet skills. The present study revealed that two-thirds of the participants possessed proficient internet skills, and those with better skills exhibited a significantly more intention to adopt the mHealth app.

Further exploration of the qualitative components revealed an additional theme for this study, which resolves the key features of the mHealth app for stroke caregiving. To ensure that this app is valuable and sustainable, the participants mentioned the must-have features for the app developer to incorporate: (i) availability of services for caregivers; (ii) provision of knowledge skills; and (iii) support for caregivers in managing stroke patients, which are closely related to the first theme: mHealth app acceptability and expectations.

Regarding the availability of services for caregivers, the features mentioned by the study participants were comparable to those in previously published reviews (17, 18). However, they emphasized the need for a healthcare service directory to connect caregivers with essential assistance, such as ambulance and home care services, as well as the importance of financial support due to significant expenses related to patient wellbeing, which is consistent with the findings of a local study on stroke management mobile applications for informal caregivers (49).

For the provision of knowledge skills, besides stroke recovery and exercise, participants also felt it was essential to include information on stroke awareness and the caregivers’ role. This is because the disease remains the third most prevalent cause of death and disability throughout the years, there is an increasing number of young stroke patients in Malaysia, and there is a lack of caregiver contribution recognition (1, 50–52). To support caregivers in managing stroke patients, as in previous studies, the integration of appointment scheduling, progress monitoring, and to-do lists was among the features to ensure and promote caregiver engagement (17, 18).

## 5. Limitations

This study has several limitations, which provide opportunities for future research. First, because the availability and accessibility of stroke-related services vary across different regions of the country, selecting healthcare providers from the northeast peninsula may restrict the generalizability of the findings to represent all healthcare providers in Malaysia. Nevertheless, the stroke clinical practice guidelines followed by healthcare providers for stroke management are standardized throughout the country; hence, findings from interviewing northeastern healthcare providers are still relevant and beneficial to targeted users.

Additionally, the present study revealed an uneven demographic distribution of healthcare providers in the northern peninsular region; the higher proportion of women shown is consistent with the gender proportion of healthcare providers in Malaysia (53). Nonetheless, given that our research is primarily aimed at evaluating the feasibility of the mHealth app, our findings remain pertinent and valid.

Finally, when using an online platform, it can be challenging to avoid sampling problems such as self-selection bias (54, 55). However, several measures were employed to ensure the validity of the interview process and to complement the qualitative findings with quantitative research to enhance the robustness of our results.

## 6. Future implication

This research contributes evidence to the implementation of digital healthcare transformation, aligning with the Malaysia 12th National Plan 2021–2025 (12MP), which aims toward the digitalization of healthcare (56). Over time, with the high acceptability of healthcare providers adopting the mHealth app as part of stroke management to assist stroke caregivers, it has the potential to enhance caregivers’ confidence and competence in managing stroke survivors. Ultimately, this will improve the quality of life for both stroke survivors and their caregivers. This development is a step toward transforming healthcare and making it more efficient for everyone concerned.

## 7. Conclusion

The findings revealed that most healthcare providers are looking forward to integrating this mHealth intervention into their patient care plans, owing to the key features identified in this study. However, to ensure the long-term sustainability of this intervention, future research should focus on determining the feasibility of integrating smartphone applications into the healthcare system after their development has been completed.

## Data availability statement

The raw data supporting the conclusions of this article will be made available by the authors, without undue reservation.

## Ethics statement

The studies involving humans were approved by Malaysia Research Ethics Committee (MREC Ref: KKM/NIHSEC/P20-922), the Human Research Ethics Committee of USM (JEPeM: USM/JEPeM/20010031), and London School of Hygiene & Tropical Medicine (LSHTM) Research Ethics Committee (LSHTM Ethics Ref: 19079). The studies were conducted in accordance with the local legislation and institutional requirements. The participants provided their written informed consent to participate in this study.

## Author contributions

KM and SK supervised the project. NS, KM, SK, TT, and IP contributed to the conception and design of the study. NS, MR, KM, and KI contributed to the investigation. NS, MM, XC, and KM performed the statistical analysis. NS, KM, SK, TT, and IP conducted the thematic analysis. NS wrote the first draft of the manuscript. All authors contributed to the manuscript revision, read, and approved the submitted version.
