# Supplementary material for: A mixed-methods study on the implementation of a mobile health application (mHealth app) for stroke caregivers in Malaysia: healthcare providers’ perspective
Source: Front Neurol. 2023 Oct 12;14:1222260. doi: 10.3389/fneur.2023.1222260 (PMC10613485; doi:10.3389/fneur.2023.1222260)
Supplement: Supplementary file 1 [file Table_1.DOCX]

Supplementary Material

A Mixed-Methods Study on the Implementation of a Mobile Health Application for Caregivers of Stroke Survivors in Malaysia: Healthcare Providers' Perception and Expectations

**Norsima Nazifah Sidek^1,2^, Tengku Alina Tengku Ismail^1^, Sureshkumar Kamalakannan^3,4^, Chen Xin Wee^5^, Muhammad Hibatullah Romli^6^, Mohamad Zarudin Mat Said^7^, Iliatha Papachristou Nadal^8^ , Khairul Azmi Ibrahim^9^, Kamarul Imran Musa^1*^**

^1^ Department of Community Medicine, School of Medical Sciences, Universiti Sains Malaysia, Kota Bharu, Kelantan, Malaysia

^2^ Clinical Research Centre, Hospital Sultanah Nur Zahirah, Kuala Terengganu, Terengganu, Malaysia

^3^ Department of Non-communicable Disease Epidemiology, London School of Hygiene and Tropical

Medicine, London, U.K.

^4^ Department of Social Work, Education, and Community Well-being, Northumbria University, Coach

Lane Campus, Newcastle Upon Tyne, U.K

^5^ Department of Public Health Medicine, Faculty of Medicine, Universiti Teknologi MARA, Sungai Buloh, Selangor, Malaysia

^6^  Department of Rehabilitation Medicine, UPM Teaching Hospital, Faculty of Medicine and Health

Sciences Malaysian Research Institute on Ageing (MyAgeing^TM^), Universiti Putra Malaysia,

Serdang, Selangor, Malaysia

^7^ Disease Control Unit, Hilir Perak District Health Office, Teluk Intan, Perak, Malaysia

^8^ Division of Care in Long Term Conditions, King’s College London, London, U.K.

^9^ Department of Medicine, Hospital Sultanah Nur Zahirah, Kuala Terengganu, Terengganu, Malaysia

*** Correspondence:**[drkamarul@usm.my](mailto:drkamarul@usm.my)

# Supplementary Table 1 : Overall construct validity and internal consistency reliability

| Domain | Mean (SD) | Factors Loading | Cronbach's alpha | Overall Cronbach's alpha |
| --- | --- | --- | --- | --- |
| **PERCEIVED_USEFULNESS** | | | | |
| 1. Improves the care | 5.39 (1.53) | 0. 727 | 0.973 | 0.979 |
| 2. More productive | 5.29 (1.51) | 0. 783 |  |  |
| 3. More effective | 5.32 (1.53) | 0. 737 |  |  |
| 4. Beneficial to job | 5.50 (1.45) | 0. 788 |  |  |
| 5. Provide care to patients more quickly | 5.44 (1.47) | 0. 782 |  |  |
| 6. Easier to provide care to patients | 5.49 (1.40) | 0. 763 |  |  |
| **PERCEIVED_EASY_TO_USE** | | | |  |
| 7. Clear and understandable | 5.16 (1.37) | 0. 620 | 0.965 |  |
| 8. Requires little effort for me | 5.01 (1.37) | 0. 823 |  |  |
| 9. Easy to use | 5.06 (1.33) | 0. 797 |  |  |
| 10. Easily use | 5.21 (1.32) | 0. 746 |  |  |
| 11. Learning is easy | 5.16 (1.37) | 0. 728 |  |  |
| 12. Easy to become proficient | 5.19 (1.31) | 0. 717 |  |  |
| **INTENTION_TO_USE** | | | |  |
| 13. Intend to use them | 5.59 (1.41) | 0.857 | 0.971 |  |
| 14. Predict would use it | 5.62 (1.36) | 0.850 |  |  |
